# Resonant Dipolar Coupling of Microwaves with Confined Acoustic Vibrations in a Rod-shaped Virus

**DOI:** 10.1038/s41598-017-04089-7

**Published:** 2017-07-04

**Authors:** Chi-Kuang Sun, Yi-Chun Tsai, Yi-Jan E. Chen, Tzu-Ming Liu, Hui-Yuan Chen, Han-Ching Wang, Chu-Fang Lo

**Affiliations:** 1 Graduate Institute of Photonics and Optoelectronics, National Taiwan University, Taipei, 10617 Taiwan; 2 Department of Electrical Engineering, National Taiwan University, Taipei, 10617 Taiwan; 3 Faculty of Health Sciences, University of Macau, Macau, China; 4 Department of Biotechnology and Bioindustry Sciences, National Cheng Kung University, Tainan, Taiwan

## Abstract

In this letter, we treat a rod-shaped virus as a free homogenous nanorod and identify its confined acoustic vibration modes that can cause strong resonant microwave absorption through electric dipolar excitation with a core-shell charge distribution. They are found to be the n = 4N-2 modes of the longitudinal modes of the nanorods, where N is an integer starting from 1 and n is the mode order quantum number. This study was confirmed by measuring the microwave absorption spectra of white spot syndrome virus (WSSV), which is a rod-shaped virus. This is also the first study to identify the “dipolar-like” mode in a rod-shaped nano-object. Our study is not only an important step to achieve rapid and sensitive detection of rod-shaped viruses based on their microwave spectroscopic features and a non-contact method to measure the Young’s modulus of rod-shaped viruses, but also is critical to formulate an efficient epidemic prevention strategy to deactivate viruses with the structure-resonant microwaves.

## Introduction

By treating icosahedral viruses as nano-spheres, a recent study has shown highly efficient structure-resonant energy transfer from microwave to the confined acoustic vibration modes of spherical viruses^1^, which results in deactivation of flu viruses with microwave power below the IEEE standard that is safe in open public space. This resonant microwave absorption process is through the dipolar coupling of electromagnetic waves with the specific “dipolar” vibration modes of the same frequency in the spherical viruses, assisted with the inherent core-shell charge separation of the nano-sphere-like virions^2–4^. Since it is a resonant conversion of a photon into a phonon of the same energy, this energy transfer is highly efficient and thus provides a new strategy for virus epidemic prevention in open public. However not all viruses are nano-sphere-like, it is thus highly desirable to explore the “dipolar-like” resonant energy transfer effect in other viruses. Besides the spherical shapes, the rod-like shape is the other common shape for viruses. Up to now, there is no study to identify the “dipolar-like” mode in a rod-shaped nano-object. In this letter, by treating a rod-shaped virus as a free homogenous nanorod, we experimentally identify the confined acoustic vibration modes that can cause strong resonant microwave absorption. They are found to be the longitudinal modes of the nanorods with a mode order quantum number n equivalent to 4N-2, where N is an integer starting from 1. This theoretical analysis was confirmed by measuring the microwave absorption spectra of the white spot syndrome virus (WSSV), which is a rod-shaped virus. Our study is not only an important step to achieve rapid and sensitive detection of rod-shaped viruses and provides a non-contact method to measure their elastic properties, but also is critical to formulate an epidemic prevention strategy to deactivate rod-shaped viruses with efficient structure-resonant microwaves.

In 1882, H. Lamb^5^ proposed the theory of confined vibration modes in spherical particles derived from a stress-free boundary condition. The confined acoustic modes of spherical nano-particles were not observed until 1986^6^. In 1992, E. Duval^7^ proposed the selection rules for far-infrared and Raman transitions of a solid sphere. According to selection rules, only dipolar modes, which are the spheroidal modes with *l* = 1 where *l* is the angular momentum quantum number, can be excited by far-infrared due to the electric-dipolar transition. However, there was no experimental observation of dipolar modes until two recent reports^2, 8^ on the terahertz (THz) absorption in TiO_2_ nanopowders and CdSe/CdTe core-shell type-II quantum dots. The type-II quantum dot study proved the existence of the resonance dipolar interaction between THz photons and the spheroidal dipolar phonons in nano-spheres. The same study also indicated the critical role of the specific core-shell spatial charge separation for the excitation of the resonance dipolar interaction. Taking advantage of the core-shell-like charge separation structure in a virus, this finding was then applied to spherical viruses by treating one icosahedral virus in serum as a single free homogeneous nano-sphere. T.-M. Liu *et al*. observed the resonance microwave absorption in spherical viruses through confined acoustic vibrations^3, 4^. The measured microwave absorption peak frequencies agreed well with those of the dipolar modes with the quantum numbers *l* = 1, n = 0 & 1, and were size-dependent of the studied viruses. This observation of resonance microwave absorption of viruses, combined with the matured microwave technology, could provide a promising physical mechanism for future development of rapid non-contact identification, quantification, manipulation, and selective destruction of viruses without the need of affinity biosensors. Recently this structure-resonant energy transfer effect from microwaves to viruses, based on the dipolar mode transition, was shown to be efficient enough so that airborne virus was inactivated with reasonable microwave power density safe for the open public^1^. This effect was demonstrated by measuring the residual viral infectivity of influenza A virus after illuminating microwaves with different frequencies and powers. Nevertheless, so far there is no experimental report related to dipolar transition through infrared/THz wave/microwave excitation of the confined acoustic phonon modes of nanorod/nano-cylinder or the rod-shaped viruses, and there is no study reported on the dipolar–like modes of rods or cylinders. It is thus highly desirable to identify the “dipolar-like” confined acoustic phonon modes in rod-shaped viruses not just for fundamental physics studies, for the non-contact assessment of the virus Young’s modulus, but also for its potential applications on virus deactivation.

The vibrations of a cylinder have been studied with a long history. In 1876, L. Pochhammer^9^ researched the vibrations of solid cylinders, and in 1886 C. Chree^10^ studied infinitely long circular rods with traction-free circumferential boundaries. In 1964, G. W. McMahon^11^ published his experimental results for natural frequencies of solid cylinders with free boundaries. In 1972, J. R. Hutchinson^12^ studied the axisymmetric free vibrations of finite length rods using a series solution to the three-dimensional theory of elasticity and further^13^ included nonaxisymmetric vibrations of a solid rod in 1980. Based on previous researches, recently H. R. Hamidzadeh and R. N. Jazar^14^ categorized the confined vibrational modes of a cylinder as follows: 1) breathing mode, where any point on the cross section of the cylinder vibrates harmonically in its radial direction; 2) torsional mode, associated with transverse displacement of the circumference of the cylinder; 3) axial/longitudinal/extensional mode, associated with axial vibration; 4) bending and axial shear/transverse mode, where the cross section of the cylinder remains the same; 5) lobar mode, where both radial and transverse displacements vary with sin(nθ)^14^. These modes have been found to be the dominant vibration modes of a cylinder or a rod-like object. The analysis of the motion for any point in a cylinder can be approximated by summing the effects of the dominant modes.

**Figure 1:**
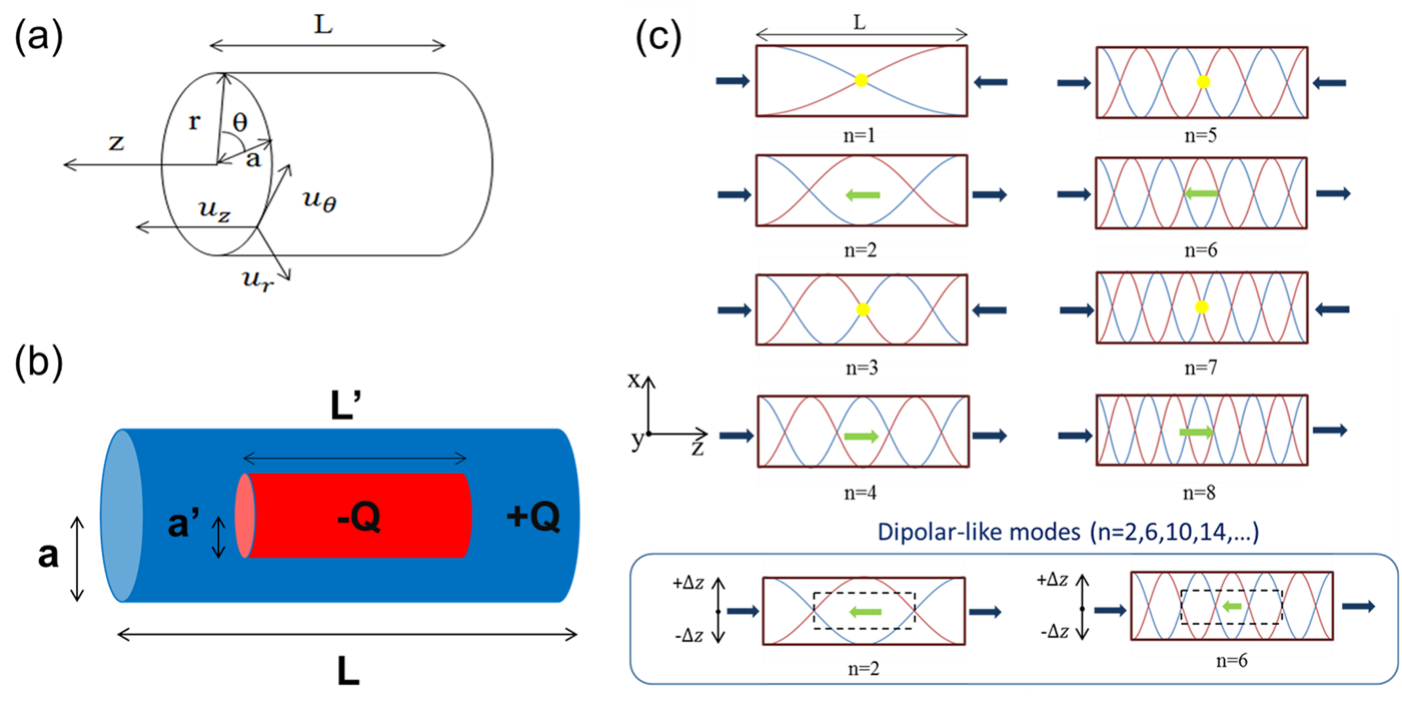
Longitudinal standing waves in a nano-cylinder. (**a**) Coordinate of a nano-cylinder; L: length of cylinder; a: radius of cylinder. ${u}_{r},{u}_{\theta },{u}_{z}\,{\rm{are}}\,{\rm{displacement}}\,{\rm{of}}\,{\rm{r}},{\rm{\theta }},{\rm{z}}$. (**b**) Schematic showing the core (red part) and shell (blue part) structure of a nano-cylinder with different charges. **a** is the radius of the whole cylinder, and **L** is the length of the whole cylinder. **a’** is the radius of core, and **L’** is the length of core; Dimension ratio = **L’/L** = **a’/a**. (**c**) Displacement of the longitudinal standing waves in a nano-cylinder. Brown and light blue curves represent the distribution of displacement when both ends have largest displacement. Dark blue and light green arrows indicate the direction of displacement at ends and center of the cylinder, respectively.

In this study, we apply the above-mentioned free boundary condition approximation to rod-like viruses in serum. After examining all different modes, we found that (as shown in Fig. 1) the longitudinal modes with the mode order quantum number (or overtone index) *n* equivalent to 4N-2 could be corresponding to the dipolar-like mode in a nano-rod for the microwave excitation under a core-shell charge separation structure, where N is an integer starting from 1. Figure 1(c) simply illustrates the displacement of the longitudinal standing waves in a nano-cylinder, where brown and light blue curves represent the distribution of displacement when both ends have largest displacement. Dark blue and light green arrows respectively indicate the direction of displacement at ends and center of the cylinder. For the mode order n = 1, 3, 5, 7, the center of cylinder becomes a node of standing waves, which is indicated by a solid yellow circle. As a result, only longitudinal modes with a mode order n = 4N-2 moves the core shell charges in the opposite directions, where N is an integer starting from 1. The confined vibration of these n = 4N-2 modes should result in dipolar coupling and is thus considered dipolar-like.

To explore the electric dipolar transition with confined acoustic vibrations in a rod-like object, here we assume that the nanorod (or cylinder) is isotropic, solid and elastic. The radius (*a*) of the cylinder is much smaller than that of length (*L*), and the boundary conditions are free. Based on the equation of motion of a three-dimensional elastic body^14, 15^

1 $${\rm{\rho }}\frac{{\partial }^{2}\overrightarrow{D}}{\partial {t}^{2}}=(\lambda +\mu )\overrightarrow{\nabla }(\overrightarrow{\nabla }\cdot \overrightarrow{D})+{\rm{\mu }}{\nabla }^{2}\overrightarrow{D},$$

where $\overrightarrow{{\rm{D}}}$ is the displacement vector, the two parameters μ and λ are known as Lamé’s constants, ρ is the mass density, and by taking displacement *u* in the 3 cylindrical coordinates r, θ, z with the form ${u}_{r}={U}_{r}{e}^{i(kz+\omega t)},\,{u}_{\theta }={U}_{\theta }{e}^{i(kz+\omega t)},\,{u}_{z}={U}_{z}{e}^{i(kz+\omega t)}$, as shown in Fig. 1(a) under the condition that only longitudinal modes are sought, the vibration equations can be derived based on the cylindrical coordinates as

2 $${\rm{\rho }}\frac{{\partial }^{2}{u}_{r}}{\partial {t}^{2}}=(\lambda +2\mu )\frac{\partial {\rm{\Delta }}}{\partial r}-\frac{2\mu }{r}\frac{\partial {\omega }_{z}}{\partial \theta }+2\mu \frac{\partial {\omega }_{\theta }}{\partial z},$$

3 $${\rm{\rho }}\frac{{\partial }^{2}{u}_{\theta }}{\partial {t}^{2}}=(\lambda +2\mu )\frac{1}{r}\frac{\partial {\rm{\Delta }}}{\partial r}-2\mu \frac{\partial {\omega }_{r}}{\partial z}+2\mu \frac{\partial {\omega }_{z}}{\partial r},$$

4 $${\rm{\rho }}\frac{{\partial }^{2}{u}_{z}}{\partial {t}^{2}}=(\lambda +2\mu )\frac{\partial {\rm{\Delta }}}{\partial z}-\frac{2\mu }{r}\frac{\partial }{\partial r}(r{\omega }_{\theta })+\frac{2\mu }{r}\frac{\partial {\omega }_{r}}{\partial \theta },$$

where

5 $${\rm{\Delta }}=\frac{1}{r}\frac{\partial (r{u}_{r})}{\partial r}+\frac{1}{r}\frac{\partial {u}_{\theta }}{\partial \theta }+\frac{\partial {u}_{z}}{\partial z},$$

and

$$2{\omega }_{r}=\frac{1}{r}\frac{\partial {u}_{z}}{\partial \theta }-\frac{\partial {u}_{\theta }}{\partial z},$$

$$2{\omega }_{\theta }=\frac{\partial {u}_{r}}{\partial z}-\frac{\partial {u}_{z}}{\partial r},$$

$$2{\omega }_{z}=\frac{1}{r}(\frac{\partial (r{u}_{\theta })}{\partial r}-\frac{\partial {u}_{r}}{\partial \theta }).$$

${\omega }_{r},{\omega }_{\theta },{\omega }_{z}\,\mathrm{should}\,\mathrm{satisfy}\,\frac{1}{r}\frac{\partial (r{\omega }_{r})}{\partial r}+\frac{1}{r}\frac{\partial {\omega }_{\theta }}{\partial \theta }+\frac{\partial {\omega }_{z}}{\partial z}=0$. By following Love’s^15^ treatment by assuming *U*_*θ*_ = 0, *L* ≫ *a*, and to solve Eq. 2 and Eq. 4, one can find the simplified solution for the confined longitudinal (axial, or extensional) vibration mode as

$${u}_{r}\propto \,\sin (\frac{n\pi z}{L})\cos ({\omega }_{n}t+\vartheta ),$$

$${u}_{z}\propto \,\cos (\frac{n\pi z}{L})\cos ({\omega }_{n}t+\vartheta ),$$

6 $${\omega }_{n}=\frac{n\pi }{L}\sqrt{\frac{E}{\rho }}=\frac{n\pi }{L}{V}_{L},$$

where ϑ is a phase constant, E is Young’s modulus, V_L_ is the longitudinal sound velocity, *ω*_*n*_ is the angular frequency of the nth mode, and *n* is the quantum number of the longitudinal mode order.

The cores of most viruses have inherent negative charges due to the phosphate groups in genomes. In contrast, the amino acids of viral capsids or envelopes have complex charge distributions on the surface. Previous studies^3, 4^ have indicated that the negatively-charged genome core and the positively-charged capsid shell of a spherical virus provide the inherent charge separation required for the activation of the electric dipolar transition with microwave resonant absorption. With a similar core-shell charge separation in rod-shaped viruses, the corresponding electric dipolar transition will occur when the confined acoustic vibration moves the core shell charges in the opposite directions and thus modify their dipole moments. As shown in Fig. 1(b), by assuming a core-shell charge separation in a rod-like virus, we have analyzed the axial displacement *u*_*z*_ of the longitudinal standing waves in a cylinder for confined longitudinal vibrational modes with different quantum number of the longitudinal mode order. By checking the direction of the axial displacement in the very center and at both ends, we found that the modes with n = 2, 6, 10, 14, … would provide the much required opposite direction oscillation between the core and shell charges, cause the dipolar coupling, and thus act as “dipolar-like” modes.

**Figure 2:**
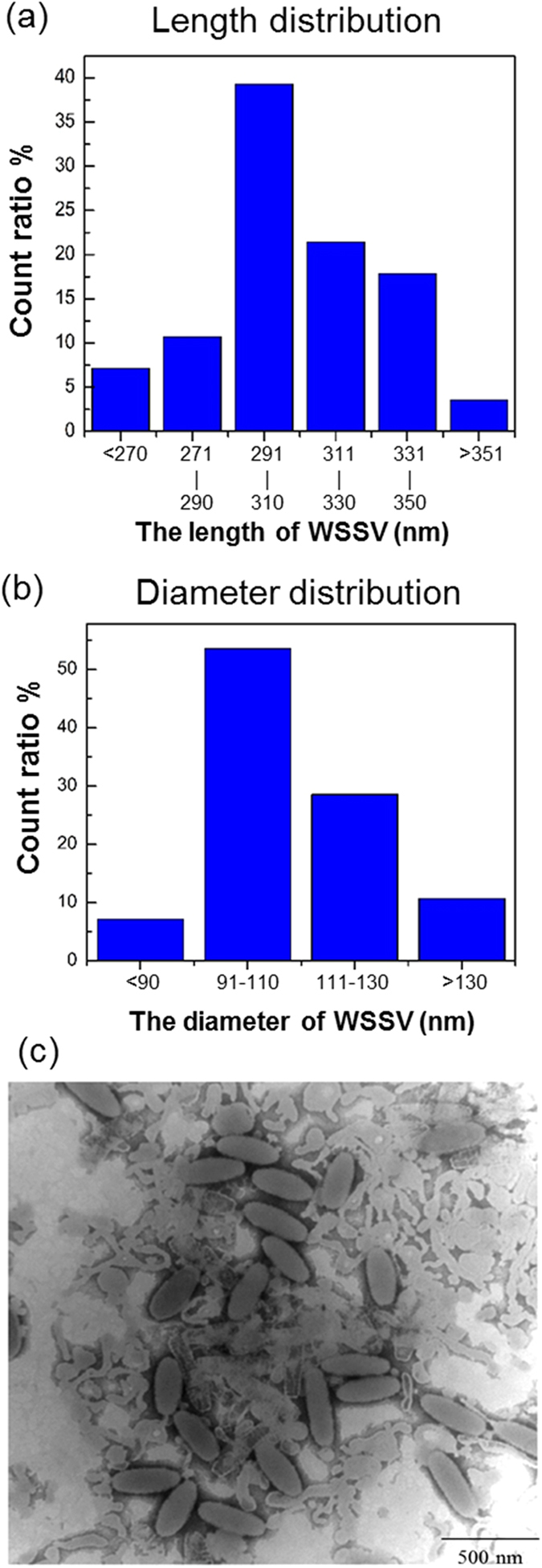
White spot syndrome virus. The (**a**) length and (**b**) diameter distribution of white spot syndrome virus (WSSV) as summarized by its (**c**) transmission electron micrographs.

For experimental verification, we chose white spot syndrome virus (WSSV). WSSV is a widespread disastrous viral pathogen of cultured shrimps and causes high economic losses. WSSV also attacks crabs, crayfish, and many other crustaceans^16, 17^. WSSV is a rod-shaped, enveloped, large double-stranded DNA virus, and the size of the enveloped viral particles have been reported to be approximately 250~380 nm in length and 80~120 nm in diameter (Fig. 2)^16, 18–21^. The mass density of a WSSV complete virion is known as 1.22 g/cm^3^ through isopycnic centrifugation in CsCl^20^, and that of the WSSV nucleocapsid is 1.31 g/cm^3^. The size of the viral DNA of around 300 kbp is above the range (100 ± 180 kbp) of baculovirus genomes^18, 19^. To predict the microwave absorption peak frequency with the electric dipolar excitation through the confined “dipolar-like” modes, one needs the effective longitudinal sound velocity *V*_*L*_ of WSSV. Recently based on Brillouin scattering measurement, the effective *V*_*L*_ of the wet Satellite Tobacco Mosaic Virus (STMV) was determined to be 1920 m/s^22^. With more genomes packed inside the capsid, the effective *V*_*L*_ of the EnteroVirus 71 (EV71) was determined to be 2450 m/s^3^ by using microwave resonant absorption spectroscopy. Based on Eq. (6), the confined longitudinal mode frequency for mode number n = 2 should be around 6.4–8.2 GHz, if the effective *V*_*L*_ is between 1920–2450 m/s. As for other mode numbers including n = 6 and 10, the expected frequencies will then be around 19.2–24.5 GHz and 32–40.8 GHz, respectively.

**Figure 3:**
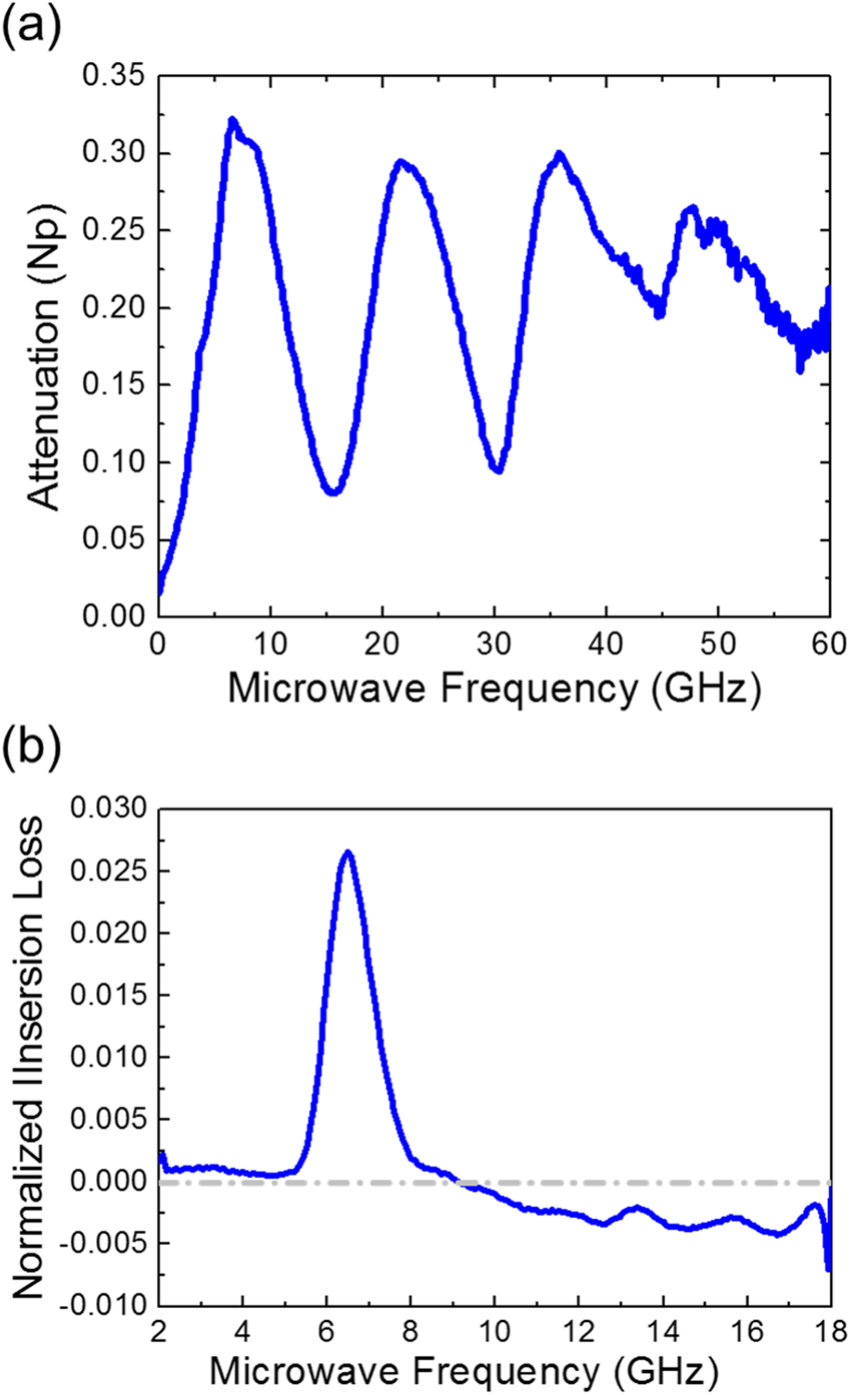
Microwave resonant absorption spectra of WSSV after removing the contributions from the background. (**a**) First set of measurement with a 65 GHz measurement bandwidth. (**b**) Second set of measurement with a 18 GHz measurement bandwidth.

The microwave absorption spectroscopy of the first set of WSSV virion sample was performed by adopting the same microwave absorption measurement system as described in ref. 3 with a wide bandwidth CPW circuit and a 65 GHz network analyzer. 1 μl of the first set of WSSV sample was uniformly dropped by a micropipette on the midst of the CPW circuit. Thus obtained microwave absorption spectra 4 minutes after was shown in Fig. 3(a). The microwave absorption spectrum shows strong absorption peaks at 6.6 GHz, 21.6 GHz, 35.7 GHz, and 47.8 GHz, with a frequency ratio close to 1:3:5:7. As predicted by our proposed model on the rod-shaped virus, the peak frequency ratio 1:3:5:7 corresponds well to the dipolar-active confined longitudinal acoustic mode order n = 2, 6, 10, and 14, respectively. These results confirmed our analysis that not all orders of the confined longitudinal vibration modes in a rod-like structure can induce dipolar transition with microwave resonant absorption. As illustrated in Fig. 1(c), only standing waves with the mode order n equal to 4N-2 have relative core-shell motion, where N is an integer starting from 1. It is important to notice that the selection rule of the high-order-acoustic-mode absorption of microwave through dipolar transition varies with the shape of viruses^3^. For spherical viruses, dipolar transition with resonant microwave absorption is through the excitation of *l* = 1 dipolar modes with n = 0 and 1 with a peak frequency ratio of 1:2, very different from the 1:3:5:7 ratio of the rod-like viruses.

**Figure 4:**
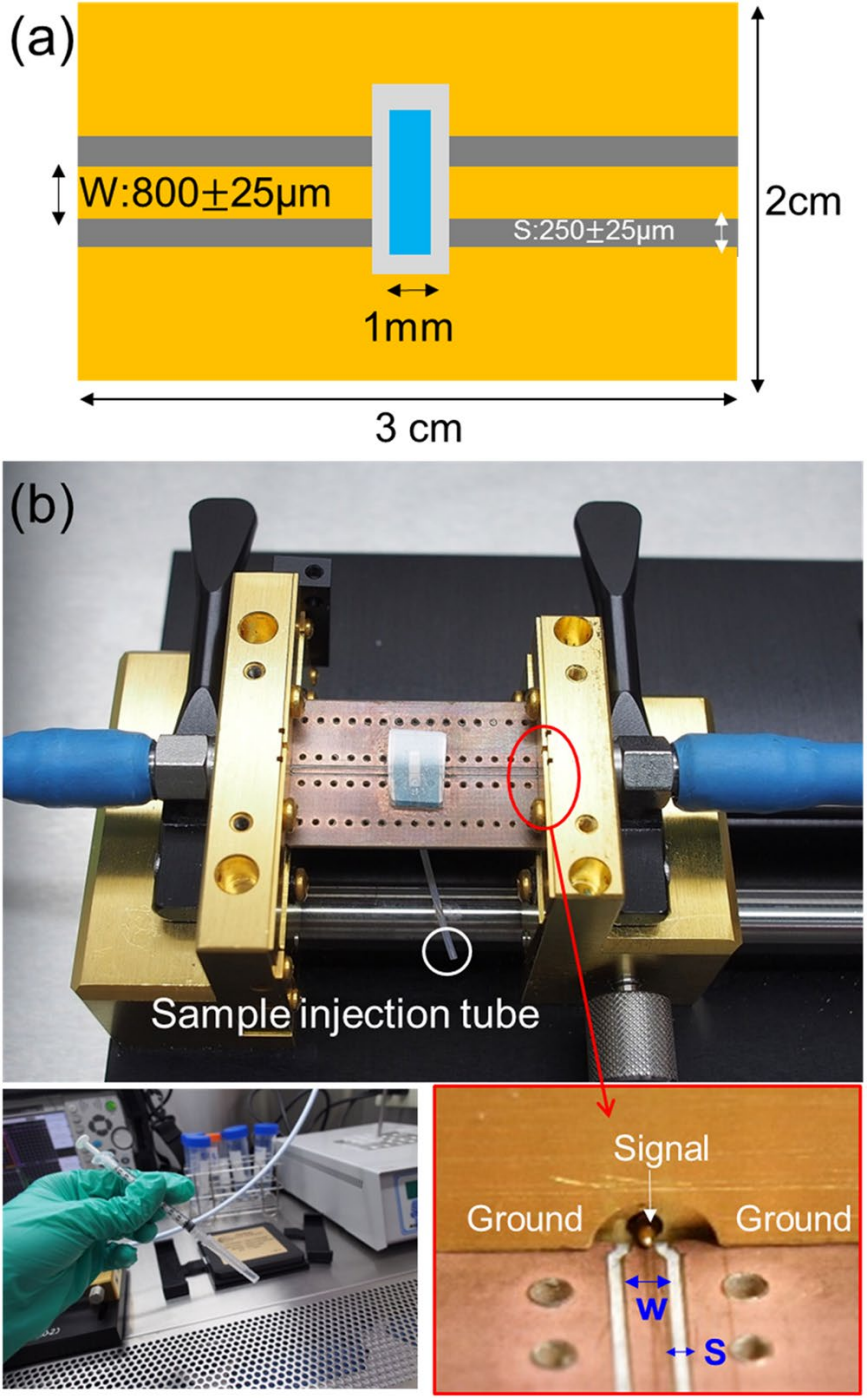
Microwave absorption spectrum measurement. (**a**) Schematic top view of a microfluidic channel sticking on top of a coplanar waveguide (CPW). (**b**) Photos showing the microfuidic channel integrated CPW on the test fixture, the 1 ml syringe, and details regarding the contact between CPW and test fixture.

The measurement on microwave absorption spectra indicated a wide bandwidth and low quality factor *Q* of the resonance modes. In a previous microwave absorption study on *Perina nuda* viruses (PnV)^4^, it was found that the hydration levels on the capsid surface of viruses can affect the bandwidth of microwave resonant absorption induced by the confined acoustic vibrations. This was evidenced by a pH dependent study, where the enhancement of the surface hydrophilicity resulted in a rise of *Q*. It is thus possible that this wide bandwidth is related to the hydration levels on the capsid surface of viruses. Another factor which can affect the bandwidth will be the purity of the samples, which would result in inhomogeneous broadening of the resonance linewidth. It was known that host-factor (vitellogenin and hemocyanin) contaminations were frequently observed on WSSV virions^16^. Hemocyanin is a host factor present in shrimp hemolymph which involved in host immune response against pathogen. To avoid possible host factor contamination to affect the measured bandwidth, we revised our purification method and prepared the second set of WSSV virion samples. To provide a better spectral resolution and to investigate the possible bending mode issue, to be discussed later, we thus employed a vector network analyzer with a microwave measurement frequency range only up to 20 GHz but with a 0.1 GHz frequency resolution. The microwave absorption spectroscopy of the second set of WSSV virion samples was performed in a biosafety cabinet while the sample temperature was kept at 25 °C, by using a microfluidic channel adhered on a coplanar waveguide (CPW) circuit and a portable vector network analyzer (Anritsu MS2028C) with a bandwidth up to 20 GHz, as described in Methods and as shown in Fig. 4. By comparing the microwave attenuation spectra of buffer solution with and without the WSSV virions, the microwave attenuation spectrum of the WSSV sample can be obtained as shown in Fig. 3(b). A sharp absorption peak at the microwave frequency of 6.5 GHz with a bandwidth of 1.2 GHz (Q = 5.3) can be observed, in good agreement with the expected n = 2 confined longitudinal mode frequency (6.4–8.2 GHz) and in agreement of the broadband measurement shown in Fig. 3(a). Our study on the second set of samples confirms the contribution of host-factor contaminations on the linewidth broadening observed in the first set of samples. The observed clean spectrum with a single 6.5 GHz absorption peak also indicates negligible dipolar interaction, when compared with the n = 2 longitudinal mode, with other confined acoustic vibrational modes including breathing modes and bending modes whose frequencies also fall into the range of our measurement. This observation also rules out the other possible microwave dipolar interaction with n = 1, 3, 4, 5 longitudinal modes of which the mode frequency should be lower than 18 GHz.

**Figure 5:**
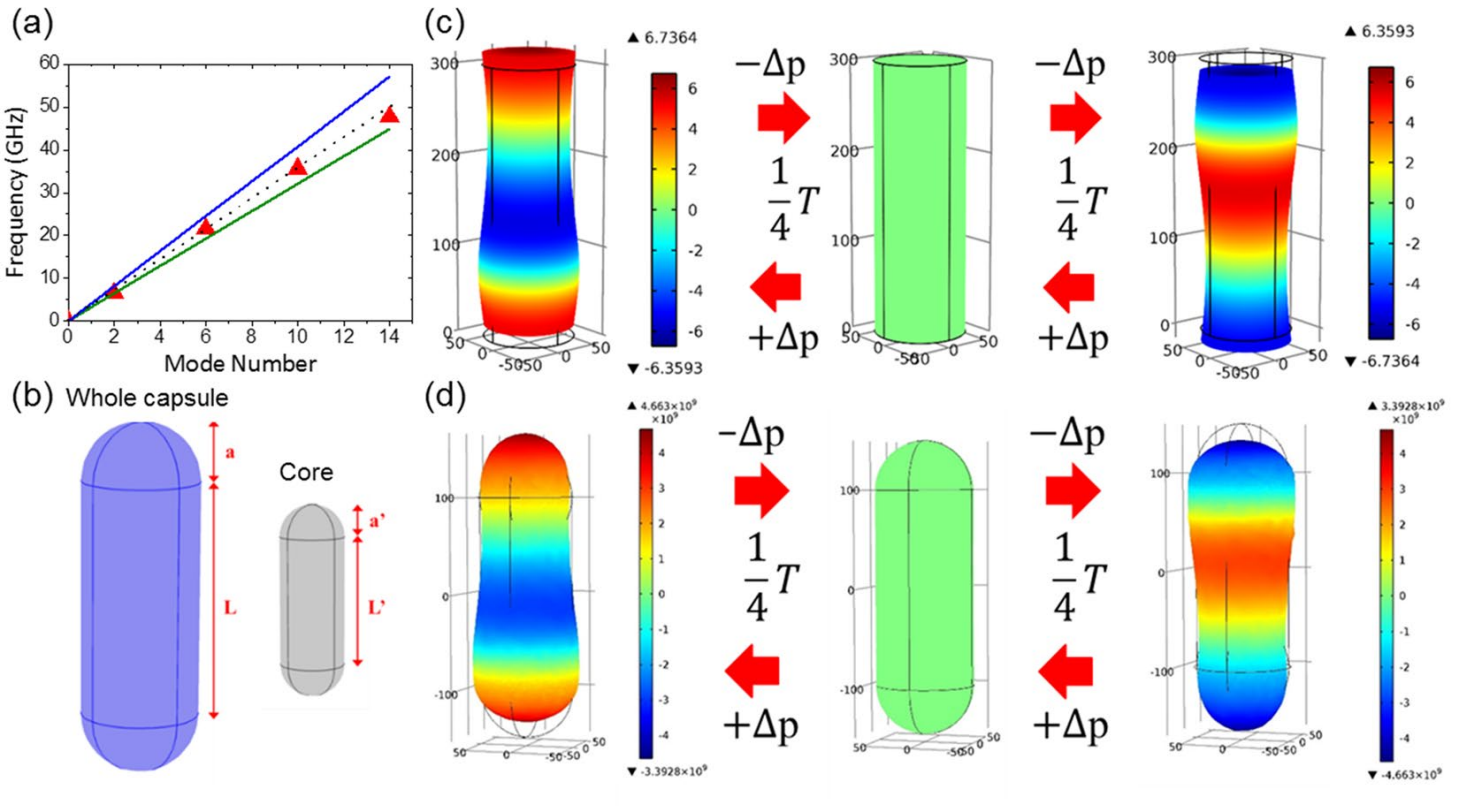
Longitudinal modes and vibrational frequencies. (**a**) A figure showing the measured resonant microwave absorption frequency (red solid triangles) as a function of the longitudinal mode order. Relationships based on Eq. 6 are shown in blue and green lines, assuming longitudinal sound velocities of 1920m/s and 2450 m/s respectively. The dot dash line shows the relationship with a longitudinal sound velocity of 2100 m/s. (**b**) Schematic showing a nano-capsule and its inner core. a is the radius of the capsule and hemispheroid, L-2a is the length of the center cylinder; and L is the total length of the capsule. Similarly, a’ is the radius of core, and L’ is the length of core. (**c**) The FEM simulation result of the n = 2 longitudinal mode vibration of a nano-cylinder with a 0.5 dimension-ratio between the core and shell. Color bar represents the displacement field. T: Period; ${\rm{\Delta }}p$: difference of dipole moment between the balance state and the maximum compression/extension state. (**d**) The FEM simulation result of the n = 2 longitudinal mode vibration of a nano-capsule with a 0.5 dimension-ratio between the core and shell.

Figure 5 summarizes the measurement result and compares the results with Eq. 6 assuming different longitudinal sound velocities. A simple fitting indicates that the effective longitudinal sound velocity of WSSV should be around 2100 m/s. With the published effective mass density of the complete WSSV virion as 1.22 g/cm^3^ ^20^, the effective Young’s modulus of WSSV can be estimated to be ~5.4 GPa. To check on the shape effect, we have developed a capsule model and to compare the effect of the capsule shape (versus a cylinder) to the measured longitudinal mode frequency. As shown in Fig. 5(b), this model comprises a cylinder and two hemispheroids. The radius of the cylinder is assumed to equal the radius of the hemispheroids. COMSOL Multiphysics was chosen as our simulation software for the solid mechanics simulation by the finite element method (FEM). We first constructed a homogenous, solid, isotropic cylinder model as shown in Fig. 1 and then compared the simulated resonant frequency of the n = 2 longitudinal mode with that simulated with a capsule shape as shown in Fig. 5(c) and (d). A 9% higher mode frequency was found in the capsule model when we input the WSSV parameters. This result indicates a modification on the estimated effective sound velocity (9% lower) and a modification on the estimated elastic constant (18% lower). This would make the effective sound velocity lower to 1920 m/s with an effective elastic constant of 4.5 GPa for capsule-like WSSV. Previously reported elastic constants on the empty soft viral tubes and shells ranged between 1.8–0.14 GPa^23^, while packing the high-density DNA/RNA cores was known to result in shell stiffening^23^. The densely packed viral cores were hard to be approached by using the AFM nanoindentation method. Our measured relatively high elastic constant could thus be the result of the densely packed viral DNA with a size of 300 kbp inside the nucleocapsid. Please notice that our reported value is the effective value considering the whole virus structure, not just the soft capsid part. This value is almost on the order of but lower than the previously measured Young’s modulus of individual Type 1 collagen fibrils^24^, which was found to be in the range from 5 GPa to 11.5 GPa.

One might wonder the possible interaction of bending mode vibration with microwaves. Bending mode of a cylinder can also be called transverse mode or flexural mode. The bending mode is associated with no cross-sectional distortion, while bending in the axial direction occurs. The odd mode-number bending modes move the center of the rod toward x (or y) while move both ends toward −x (or −y), following the coordinate system shown in Fig. 1(c), thus could also move the core and shell charges in opposite directions. With the nano-capsule model shown in Fig. 5, we have calculated the mode frequencies of different bending modes by using COMSOL Multiphysics. After calibration with the measurement results, the n = 1, 3, 5 bending mode frequencies should be around 1.6, 5.5, and 8.3 GHz, respectively. With a limited measurement range, we are not able to verify the contribution of the n = 1 bending mode. As for other odd bending modes, the observed clean spectrum COMSOL with a single absorption peak at 6.5 GHz indicates the negligible contribution of high-order bending modes to the microwave resonant absorption.

In summary, cores of most viruses have inherent negative charges due to the phosphate groups in genomes. In contrast, the amino acids of viral capsids or envelopes have complex charge distributions on the surface. Such a core-shell charge separation structure paves the road for the dipolar coupling between microwaves and confined acoustic vibrations. The microwave resonant absorption processes would thus occur in viruses when the confined acoustic vibration moves the charges and changes their dipole moments. By treating icosahedral viruses as nano-spheres, a recent study has shown highly efficient structure-resonant energy transfer from microwave to the confined acoustic vibrations in spherical viruses, resulting in efficient deactivation of flu virus with low microwave power safe for the open republic. Except for spheres, rods and filaments are among the other shapes most commonly found in viruses. In this work, we investigated the dipolar-like confine acoustic modes in rod-like viruses with a core-shell charge separation. As a result, we found that the selection rule of the confined acoustic-mode for microwave resonant absorption varies with the shape of viruses. By using 300 nm long baculoviruses as our test target, we proved our hypothesis that longitudinal modes with a mode order quantum number n = 4N-2 can induce resonant microwave absorption. This is not only the first study to reveal the “dipolar-like” confined acoustic modes in a nano-cylinder, but also provide a non-affinity way to detect and distinguish a rod-like virus from a spherical virus. This microwave resonant absorption spectroscopy could serve as a convenient way to measure the elastic constant of a virus. Our finding will also help to formulate a strategy for non-contact low-power virus deactivation.

## Methods

### WSSV Sample Preparation

Two sets of samples were prepared in our study. For the first set of virions, following the method of Tsai *et al*.^16^, we infected healthy *Procambarus clarkii* crayfish using inoculated hemolymph prepared from WSSV-infected *Penaeus monodon*. After one week, the intact virions in the crayfish hemolymph were purified as described previously^16, 19^, by using sucrose gradient ultracentrifugation (89,000 g × g for 2 hours). The final pellet of the intact WSSV virions was dissolved in Tris buffer (50 mM Tris, 5 mM MgCl_2_, pH 7.5). Characterized by real-time PCR, the number density of the virus was 5.5 × 10^11^ genome copies/ml and 1.7 × 10^9^ genome copies/ml for the first and the second set of virion samples. From the TEM image (Fig. 2(c)), the rod-shaped WSSV virions have on average a length of 300 nm (Fig. 2(a)) and a diameter of 100 nm (Fig. 2(b)). Except for virus solution, we also prepared the same Tris buffer (without virions) for calibration measurements.

For the first set of samples, host factors (vitellogenin and hemocyanin) contaminations were observed on the SDS-PAGE gel^16^. Hemocyanin is a host factor present in shrimp hemolymph which involved in host immune response against pathogen. To avoid the host factor contamination problem to affect the analytic results, we revised our purification method according to Xie *et al*.^25^ and prepared the second set of samples. For the second set of samples, all tissues excluding hepatopancrease of infected crayfish were used for the virus purification. For the purification of the WSSV virions, hemolymph collected from WSSV-infected moribund shrimp was centrifuged to remove the hemocytes and then kept at −80 °C as the virus stock. Crayfish (Procambarus clarkii) were injected with diluted (1:500 in PBS) virus stock, and 5~9 days later, all tissues except for the organs in the cephalothorax were cut into small pieces and homogenized in TESP buffer (50 mM Tris, 5 mM EDTA, 1 mM PMSF, 500 mM NaCl, pH 8.5; 10 ml/g tissue). Instead of ultracentrifugation, the virus was first centrifuged for 5 min at 3500 × g at 4 °C, the supernatant was collected and further centrifuged at 30,000 × g for 30 min at 4 °C. The supernatant and the pink upper layer of the pellet were gently removed, and the remaining gray/white pellet was then resuspended with TM buffer (50 mM Tris, 5 mM MgCl2, pH 7.5). The purity of the WSSV virion samples was checked using SDS-PAGE (Sodium dodecyl sulfate polyacrylamide gel electrophoresis) and Western blotting, and the integrity of the virions was confirmed by negative staining TEM. By using this purification method, host factors (e.g. hemocyanin) contaminations were significantly reduced in the second set of samples.

### Microwave Absorption Spectrum Measurement

The microwave absorption spectroscopy of the second set of WSSV virion sample was measured by using a microfluidic channel adhered on a coplanar waveguide (CPW) circuit and a portable vector network analyzer (Anritsu MS2028C) with a bandwidth up to 20 GHz. This CPW circuit substrate used the proprietary woven glass reinforced ceramics (Rogers Corporation, RO4003C) as the dielectric material with copper layers on two sides. The substrate and metal layers are around 1.5 mm and 35 μm in thickness. The dielectric constant of the dielectric material is 3.33–3.43 and the dissipation (loss) factor of that is below 0.0035 at 10 GHz. A mechanical engraving approach was used to define two slots on one side of the substrate. The width of slot and signal lines were 250 and 800 μm, respectively. A simple microfluidic channel was then adhered on top of the CPW circuit (Fig. 3(a)). For microwave absorption spectrum measurement, it was performed in a biosafety cabinet while the sample temperature was kept at 25 °C through a temperature controller. By injecting the virion solution into the microfluidic channel above the CPW circuit and by measuring the microwave reflection S_11_ and the transmission S_21_ parameter of the CPW circuit, the microwave attenuation spectra can be evaluated as described previously in ref. 3. By comparing the microwave attenuation spectra of buffer solution with and without the WSSV virions, the microwave attenuation spectrum of the WSSV sample can be obtained
